# Relationships between Wasting and Stunting and Their Concurrent Occurrence in Ghanaian Preschool Children

**DOI:** 10.1155/2016/4654920

**Published:** 2016-06-09

**Authors:** Mahama Saaka, Sylvester Zackaria Galaa

**Affiliations:** ^1^Department of Community Nutrition, School of Allied Health Sciences, University for Development Studies, P.O. Box TL 1883, Tamale, Ghana; ^2^Department of Social, Political and Historical Studies, Faculty of Integrated Development Studies, University for Development Studies, P.O. Box 520, Wa, Ghana

## Abstract

*Objective*. The main aim of the study was to assess the magnitude of concurrent wasting and stunting among Ghanaian preschool children. Secondly, we investigated the relationship between wasting and stunting as well as factors associated with these conditions.* Methods*. This paper is based on reanalysis of anthropometric and other relevant data which was collected in the 2014 Ghana Demographic and Health Survey. The data set consisted of 2,720 preschool children aged 0–59 months. We conducted three-step moderated hierarchical multiple regression analyses to determine independent predictors and moderators of height-for-age *Z*-score.* Results*. Nationally, the prevalence of concurrent wasting and stunting among children aged 0–59 months was low at 1.4% but it varied geographically with the Upper East Region having the highest prevalence of 3.2% (95% CI: 1.7–5.8). Children who had low weight-for-height *Z*-scores were at a higher risk of linear growth retardation (stunting) especially among children aged less than three years. A 1-unit increase in weight-for-height *Z*-score (WHZ) was associated with 0.07 standard units' increase in height-for-age *Z*-score (HAZ) [*β* = 0.071 (95% CI: 0.03, 0.15)].* Conclusions*. The study results suggest that weight-for-height relates to linear growth but this relationship is moderated by age of child. Stunting and wasting share some common risk factors. Therefore, measures to prevent wasting may positively influence linear growth.

## 1. Introduction

Undernutrition is associated with 2.2 million child deaths and 21% of disability-adjusted life years lost and more than 178 million children are stunted in developing countries [1]. Therefore, promoting and maintaining nutritional well-being cannot be underestimated as we forge ahead with the Sustainable Development Goals (SDGs). Aside from being a major risk factor for child mortality, childhood undernutrition has the long-term effects that include lower attained schooling, decreased economic potential, and chronic illness in adulthood [2–4].

Child undernutrition in the forms of wasting (weight-for-height *Z*-score < −2) and stunting (height-for-age *Z*-score < −2) continues to be a burden especially in the developing countries [1, 5]. The causes of undernutrition are diverse but in most cases include limited quality or quantity of food, suboptimal feeding practices, and high rates of infectious diseases [6, 7]. Acute undernutrition (wasting) occurs as a consequence of short-term response to inadequate intake or an infectious disease episode [8] and can be reversed if the child has access to adequate dietary intake in an environment that is free from infectious disease [9]. On the other hand, stunting results from inadequate intake of food over a long period of time that may be worsened by recurrent infections [10]. It is suggested, however, that although wasting is a short-term health issue, repeated episodes of it may lead to stunting [11, 12]. This is perhaps what happens in many resource-poor settings, where dietary intake is consistently inadequate and infectious diseases are highly prevalent. Under such circumstances, catch-up growth may be restricted thereby resulting in persistent high proportion of children being stunted [9].

The prevalence of stunting has decreased globally but this has not been consistent in all regions of the world. Stunting in Africa remains relatively unchanged [13]. The most recent Ghana Demographic and Health Survey, conducted in 2014, showed that the prevalence of stunting among preschoolers has decreased from 28% in 2008 to 19% in 2014 [14]. However, there are great regional disparities, with Northern Region having the highest prevalence of 33.1%.

Though information is available on the prevalence and factors associated with chronic and acute undernutrition in many countries including Ghana, there is little information on the magnitude and factors that contribute to the development of concurrent stunting and wasting within the same children. Furthermore, the relationship between the two forms of undernutrition in Ghana has not been documented.

In this paper, we further analyzed data from the 2014 Ghana Demographic and Health Survey (GDHS), to quantify the extent of combined stunting and wasting in preschool children. The 2014 GDHS was a nationally representative survey of 9,396 women aged 15–49 and 4,388 men aged 15–59 from 11,835 interviewed households. The data set was downloaded, with permission, from the MEASURE DHS website in SPSS format. The survey was implemented by the Ghana Statistical Service (GSS), the Ghana Health Service (GHS), and the National Public Health Reference Laboratory (NPHRL) of the GHS.

The main aim of the present study was to assess the magnitude of concurrent wasting and stunting among Ghanaian preschool children. Secondly, we investigated the relationship between wasting and stunting as well as the factors associated with these conditions in the Ghanaian population.

## 2. Materials and Methods

### 2.1. Study Design, Population, and Sampling

This study is based on reanalysis of anthropometric and other relevant data which was collected in the 2014 Ghana Demographic and Health Survey (DHS). For the current analysis, the data set consisted of 2,720 preschool children aged 0–59 months.

### 2.2. Data Collection

A nationally representative survey data was obtained by trained data collectors according to DHS fieldwork standard procedures, details of which can be found in the 2014 Ghana Demographic and Health Survey report [14]. In brief, stratified cluster sampling was applied whereby each region was considered a stratum. Women of childbearing age were interviewed face-to-face using structured questionnaire. Information including anthropometry and sociodemographic and infant and young child feeding (IYCF) practices was collected.

#### 2.2.1. Independent and Dependent Variables

The main outcome variable for this study was the nutritional status of the child measured as stunting, wasting, and concurrent wasting and stunting. The independent variables (predictors) for this study were maternal weight and height, reproductive factors (parity, level of education, place of delivery, and prenatal care utilization), child characteristics (e.g., sex and birth weight), malarial infection, child dietary intake, and household wealth index. A brief description of the main independent and dependent variables is as follows.

*Nutritional Status Assessment.* All children younger than 5 years of age were selected for anthropometric measurements. Weight was measured using digital portable weighing calibrated SECA 878 digital scales to the nearest 0.1 kg. The children were weighed with minimum clothing and without shoes. Weighing scale was calibrated to zero before taking every measurement.

Measurement of length (recumbent length) was done in a lying position with wooden board for children aged under two years and measurement of height for children aged above two years' stature was in a standing position in centimeters to the nearest 1 cm. The crown-heel length was taken using an infantometer (a flat wooden surface with head and foot boards). Height measurements were carried out using the infant/child Shorr Productions Measuring Board.

The anthropometric data were converted into three nutritional indices: height-for-age, weight-for-height, and weight-for-age. Classification of the nutritional status of the children was based on sex- and age-specific *Z*-scores using WHO Growth Standards [15]. Stunting and wasting were defined as *Z*-scores less than 2 (i.e., <2 standard deviation below the age-specific/sex-specific reference mean).

#### 2.2.2. Other Variables

The analyses included other several household characteristics such as household wealth, region of residence, source of drinking water, and type of toilet facilities. The wealth variable categorized respondents into quintiles according to the household's score on the DHS wealth index, which is based on the household's amenities, assets, and living conditions [16]. The other explanatory measures were whether the household was in an urban or rural area and the educational level of the mother/caretaker.

### 2.3. Statistical Analysis

The analysis of data took into account the complex design of multistage cluster surveys. All quantitative data were coded for statistical weighted analysis using SPSS Complex Samples module for windows 21.0 (SPSS Inc., Chicago). This was done in order to make statistically valid population inferences and computed standard errors from sample data.

The association between dependent variable and independent variables was determined using both multiple linear and logistic regression modeling, which included all potential confounders that were significant at *p* values < 0.05 in the bivariate analysis. The logistic regression outputs were presented as adjusted odds ratios (AOR) with 95% confidence intervals (CI).

Multicollinearity was investigated by using the variance inflation factor (VIF). A VIF (the reciprocal of the tolerance statistics) of greater than 5 is generally considered evidence of multicollinearity. In all cases, the values were less than 2.0, indicating that multicollinearity was low or absent.

Potential effect modification (statistical interaction) was investigated to ascertain whether the relationship between WHZ and HAZ was moderated by the age of the child. Effect modification was identified and adjusted for through using three-step moderated hierarchical multiple regression analyses. In the hierarchical regression analyses, control variables were entered in the first stage. This was followed by the key independent variables of interest (i.e., WHZ and age group) and finally the interaction term (moderation). The interaction term comprised the product of the centered WHZ and centered age group of the child.

Before testing for associations between independent variables and the dependent outcomes, the data were cleaned and outliers removed. *Z*-scores which were outside the WHO flags, WHZ −5 to 5; HAZ −6 to 6; and WAZ −6 to 5, were excluded from the data set.

## 3. Results

### 3.1. Sociodemographic Characteristics of the Sample

Though a total of 2782 children aged 0–59 were surveyed, 62 (2.2%) of them had missing values for the anthropometric data and were thus excluded in the final analysis.

Weighted frequencies for selected sociodemographic characteristics of the study sample are given in Table 1. The mean age of respondents was 36.8 ± 7.6 years. Nearly 34.0% of the respondents had no formal education at all and 22.2% were classified as the poorest in terms of household wealth index. With respect to occupation, petty trading (sales) and agricultural farming were common among the mothers.

### 3.2. Prevalence of Malnutrition among Children Aged 0–59 Months

In the study population, 17.9%, 4.7%, and 10.8% were stunted, wasted, and underweight, respectively. The prevalence of concurrent wasting and stunting was 1.4% (Table 2).

Nationally, the prevalence of concurrent wasting and stunting among children aged 0–59 months was 1.4% but it varied geographically with the Upper East Region having the highest prevalence of 3.2% (95% CI: 1.7–5.8) and the lowest prevalence of 0.5% (95% CI: 0.1–3.7) in the Volta Region. The proportion of children suffering from undernutrition according to age group, gender, and geographical location is shown in Table 3. The results indicate that both wasting and stunting appear to be common as early as 0–5 months in the Ghanaian population.

The global acute malnutrition (GAM) level was the highest in the younger age group aged 6–11 months. Geographically, it was the highest in the Upper East Region. For chronic undernutrition (stunting), the highest levels were found among children aged 24–35 months in the Northern Region and the lowest level was among children aged 0–5 months.

### 3.3. Relationship between WLZ and LAZ

Correlation analysis in Table 4 shows that weight-for-height/length relates to linear growth but this relationship was different according to the age group of the child. The association was the strongest among children aged 0–5 months and 12–23 months. Whereas a negative association existed among children aged under 6 months, a positive association appeared from 12 months. However, no discernible association was observed among children aged 6–11 months and among children aged 48–59 months. No association was observed in the whole sample put together.

Wasting is associated with stunting but the relationship is moderated by age of the child. Hierarchical regression analysis was used to test interactions, whereby we entered the main effects predictors before entering the interaction term. *R* ^2^ Change measures and tests the effect sizes of components, while controlling for variables that were entered into the model earlier. The interaction term added significantly beyond the main effects (*R* ^2^ Change = 0.012, *p* < 0.001), indicating that there was a statistically significant interaction between WHZ and age group in predicting HAZ.

Also, from Table 5, the interaction term was statistically significant [*β* = 0.124 (95% CI: 0.01, 0.21, *p* < 0.001)] and this confirms that the relationship between WHZ and HAZ differs according to the age group of the child. A 1-unit increase in weight-for-height *Z*-score (WHZ) was associated with 0.07 standard units' increase in height-for-age *Z*-score (HAZ) [*β* = 0.071 (95% CI: 0.03, 0.15)]. WHZ was not associated with HAZ in the absence of the interaction term.

### 3.4. Predictors of Stunting and Wasting

A host of explanatory variables were tested for their association with the outcome variables. The variables tested included age and sex of child, type of residence, current breast feeding status, diarrhoeal infection, utilization of antenatal care services, birth interval, parity, birth weight, maternal height, maternal educational level, household wealth index, source of drinking water, type of toilet facility, bottle feeding, minimum dietary diversity score, and number of children aged under five in household.

Predictors of stunting were more common than wasting and some factors were associated with both (Table 6). The greatest predictors of stunting were low maternal height, low birth weight (LBW), whether child is wasted or not, poverty, low utilization of antenatal care services, age of the child, and increased parity. These are mainly biological. The only behavioural variable that is amenable is utilization of ANC services. This set of variables accounted for 17.8% of the variability of stunting (Nagelkerke *R* Square = 0.178). For wasting, the key predictors were LBW, age of the child, wealth index, and residence in rural settings and these variables accounted for 10% of the variance in prevalence of wasting (Nagelkerke *R* Square = 0.101). The low percentage of variance accounted for in stunting and wasting suggests that a lot more variables are responsible but were perhaps not measured.

The adjusted multivariable regression model showed that LBW, age of child, and household wealth index were three significant common predictors of wasting and stunting. Compared to their counterparts who had normal birth weight (≥2.5 kg), children whose birth weight was less than 2.5 kg were about 2.0 times (AOR = 2.0 [CI: 1.10, 3.64]) and 2.7 times (AOR = 2.68 [CI: 1.67, 4.31]) more likely to be wasted and stunted, respectively.

## 4. Discussion

The main aim of the study was to assess the magnitude of concurrent wasting and stunting among Ghanaian preschool children. Secondly, we investigated the relationship between wasting and stunting in the Ghanaian population.

Several important findings emerged from this study. The prevalence of concurrent wasting and stunting among children aged 0–59 months was low at 1.4% but it varied geographically with the Upper East Region having the highest prevalence of 3.2%. Children who were wasted were at a higher risk of linear growth retardation (stunting) especially among children aged 0–5 months and 12–23 months.

### 4.1. Prevalence of Concurrent Wasting and Stunting

Nationally, the prevalence of concurrent wasting and stunting among children aged 0–59 months was low at 1.4% but it varied geographically with the Upper East Region having the highest prevalence of 3.2%. The low prevalence of concurrent wasting and stunting may be due to the fact that wasting varies greatly depending on the season the study was conducted in. Wasting and stunting are both associated with increased mortality, especially when both are present in the same child [17–19].

### 4.2. Relationship between Wasting and Stunting

The results of the current analyses show that weight-for-height relates to linear growth but the nature and strength of association were moderated by the age group of the child. There were therefore age differences in the relationship between wasting and stunting. The association was the strongest among children aged 0–5 months and 12–23 months. Whereas a negative association existed among children aged under 6 months, a positive association appeared from 12 months onwards.

In a review of eight cohort studies involving 1599 children, wasting was associated with a higher risk for linear growth retardation and that wasting after 6 months of age is associated with a lower attained length-for-age at 17 months [20]. In the same study, children who were wasted only in the first six months of life were not observed to have linear growth deficits at the end of follow-up compared with children who were never wasted. This means that children who were wasted early in life were unlikely to be stunted, perhaps due to catch-up growth.

Cross-sectional studies at the population-level have demonstrated inconsistent relationships between prevalence of stunting and wasting in childhood [21, 22]. The analysis of the association between prevalence of wasting and stunting in 22 less developed countries suggested that the association might be nonlinear [21]. Subsequent analysis concluded that different patterns emerged when continents were considered separately and that wasting and stunting are not just different presentations of the same phenomenon of dietary inadequacy, varying only in terms of timing or intensity [23].

Whereas some studies have found little or no association between wasting and stunting, others have found that these two variables were positively associated among children aged 12–23 months in Asian and Eastern Mediterranean countries [22, 24]. Longitudinal studies that explored the relationship between acquisition of weight and height at the individual level revealed that periods of the lowest linear growth follow periods of the lowest weight acquisition [11, 21, 25–29]. So there is ample evidence that suggests that ponderal growth faltering can increase the risk of linear growth faltering.

There has been no clear mechanism by which wasting may lead to stunting. Two studies that have examined the growth pattern of children recovering from severe acute malnutrition (SAM) have shown that wasting may be a cause of stunting [30, 31]. Both studies suggest that growth in height takes place only when the body has a minimum of energy reserves. This relationship is also well manifested along with seasonal changes in growth in height and weight, where children appear to grow in height only when their weight-for-height is high at the beginning of the season [26, 28].

Body fat plays a critical role in regulating bone mass and linear growth [29]. The fact that wasting is a reflection of depletion in fat and muscle masses implies that a child who is wasted may suffer from linear growth. It has been demonstrated that the fat tissues produce leptin, which has an influence on bone density and catch-up growth [32, 33]. Fat secretes multiple hormones, including leptin, which may have a stimulating effect on the immune system. Once leptin has an effect on bone growth, it is presupposed that wasting indirectly affects linear growth. This perhaps explains why wasted children who usually have low fat stores have reduced linear growth. From the foregoing, though high fat stores are needed to promote linear growth, they are not sufficient because stunting can coexist with a high overweight prevalence in some populations [34]. We do know that high fat concentration is associated with obesity. Obviously, nutrients such as sulfur, phosphorus, calcium, magnesium, vitamins D, K, and C, and copper are required for skeletal growth rather than for growth of other lean tissues [35].

In spite of the emerging relationship between wasting and stunting and their functional consequences, there remain evidence gaps and mechanisms that require confirmation. For example, the relationship between fat stores and stunting and the possible role of leptin remain inconclusive [36].

### 4.3. Predictors of Wasting and Stunting

In this study, the predictors of stunting were more common than that of wasting and may be due to the fact that the prevalence of wasting was significantly lower than that of stunting in the same population. Consequently, statistical power to detect significant associations between wasting and other variables was low. Except for type of residence (i.e., rural or urban), all the risk factors for wasting were also associated with stunting and this finding is consistent with one study which had reported that there were no risk factors for wasting that were not associated with stunting [37].

Both biological and behavioural maternal factors were strongly associated with the nutritional status of children. Stunting was positively associated with low maternal height, LBW, frequency of prenatal care, age of child, and level of household wealth index. Household wealth index was a common predictor of both stunting and wasting. Several studies have shown that children from households that fall within the poorest wealth quintile bracket were mostly likely to be malnourished, compared to their counterparts from rich households [38–40]. In India, household wealth index associated negatively with both wasting and stunting [37]. Similar studies in four countries including Ethiopia, India (Andhra Pradesh), Peru, and Vietnam show that children residing in the lowest wealth quintile households had significantly increased probabilities of being stunted in comparison to children residing in the highest wealth quintile households [41]. This goes to confirm that childhood undernutrition is strongly associated with poverty in some developing countries.

Not surprisingly, LBW (proxy for intrauterine growth restriction) was the most consistent risk factor for both stunting and wasting for all ages. Therefore, birth weight determines linear and ponderal growth. These findings concur with earlier studies that were conducted in both developed and developing countries in which low birth weight accounts for a significant proportion of the variance in stunting and wasting. For example, a number of prospective cohort studies carried out in the Cebu region of the Philippines and Ethiopia showed that prevalence of stunting at 12 months of age was significantly higher among infants who were born with low birth weight (LBW) and was shown to be a stronger predictor of intrauterine growth retardation [42–44]. An analysis of data from birth cohorts and longitudinal studies involving children aged 12–60 months from low- and middle-income countries (LMIC) concluded that childhood stunting may have its origins in the foetal period [45]. Another prospective population-based cohort study of 767 rural Malawian children from birth to 36 months of age showed that the intrauterine period and the first 6 months of life are critical for the development of stunting [46].

A similar study among urban squatter children in Karachi, Pakistan, reported that intrauterine growth retarded children had a higher risk of stunting and wasting, compared to children who were appropriate for gestational age [47]. An ecological, cross-sectional analysis of secondary data from Africa, Latin America, and Asia found LBW as a predictor of wasting prevalence in all three regions [48].

The findings in the present study support already established biological and epidemiologic evidence that stunting and wasting though representing two different processes of malnutrition [9, 11, 23, 43, 49] are positively associated. This finding supports calls that evidence-based maternal health interventions targeting this risk factor are urgently needed [47, 50].

Consistent with similar studies, increasing age of the child is associated positively with stunting but negatively with wasting [38, 51]. Our results also show that low maternal height below 145 cm (i.e., short stature) increased the risk of stunting but was not associated with wasting. This confirms earlier research findings that high maternal height [38, 52] is positively associated with improved child nutrition. The significant association between stunting and maternal factors indicates that intervention must equally focus on children and their mothers. Many experts have indeed advocated that empowering women by improving their health may prove to be one of the best approaches to promoting the health and well-being of children in the Third World [53].

In consonance with global epidemiological evidence [21, 54, 55], the present analyses indicate that wasting and stunting share some common predictors and that linear growth might still be constrained by other limiting factors that were not assessed in the current study.

One of the strengths of this study is that a large, nationally representative sample was used. However, it is not without limitations. The cross-sectional nature of the study also makes it difficult to demonstrate cause-and-effect relationships. Notwithstanding this, we have provided important insights into the relationship between wasting and stunting.

## 5. Conclusion

The present analyses indicate that the prevalence of concurrent wasting and stunting among children aged 0–59 months, though low, varied geographically. Furthermore, children who were wasted were at a higher risk of linear growth retardation (stunting) especially among children aged 12–23 months.

The risk factors associated with malnutrition in the Ghanaian population suggest that interventions that promote ponderal growth may also prevent stunting. In particular, infants born of low birth weight should be singled out for intervention. In addition, despite the inherent weaknesses of the study design, we propose that women and children in this population would benefit from interventions that increase socioeconomic status and utilization of prenatal care services.

## Figures and Tables

**Table 1:** Sociodemographic characteristics of sample (*N* = 2,720).

Characteristics	Frequency (*n*)	Percentage (%)
*Age of children (months)*		
0–5	312	11.5
6–11	288	10.6
12–23	580	21.3
24–35	552	20.3
36–47	505	18.6
48–59	483	17.8
*Gender of child*		
Male	1407	51.7
Female	1313	48.3
*Educational level of mothers*		
No education	975	35.8
Primary	565	20.8
Secondary	1085	39.9
Higher	95	3.5
*Household wealth index*		
Poorest	889	32.7
Poorer	575	21.1
Middle	516	19.0
Richer	404	14.9
Richest	336	12.4
*Occupation of respondent*		
Not working	483	17.8
Professional/technical/managerial	99	3.6
Clerical	16	0.6
Sales	849	31.3
Agricultural—self-employed	878	32.3
Agricultural—employee	24	0.9
Services	23	0.8
Skilled manual labour	320	11.8
Unskilled manual labour	28	1.0

**Table 2:** Prevalence of malnutrition among children aged 0–59 months (*N* = 2,720).

Characteristics	Mean ± SD	Frequency (*n*)	% (95% CI)
*Nutritional status*			
Height-for-age *Z*-score (HAZ)	−0.92 ± 3.0		
Weight-for-height *Z*-score (WHZ)	−0.24 ± 2.9		
Weight-for-age *Z*-score (WAZ)	−0.69 ± 2.9		
Stunting (HAZ < −2)		522	17.9 (16.1–19.9)
Severe stunting (HAZ < −3)		145	4.8 (3.7–6.1)
Wasting (WHZ < −2)		133	4.7 (3.7–6.0)
Severe wasting (WHZ < −3)		24	0.7 (0.4–1.3)
Concurrent wasting and stunting		39	1.4 (0.9–2.0)
Underweight (WAZ < −2)		297	10.8 (9.3–12.5)

^*∗*^ *Z*-scores: length-for-age, weight-for-age, and weight-for-height, WHO Standard 2006.

**Table 3:** Prevalence of undernutrition according to age, gender, and geographical location (weighted analysis).

	*N*	Stunting	Wasting	Concurrent wasting and stunting
		*n* (%)	*n* (%)	*n* (%)
*Age (months)*				
0–5	312	20 (7.2)	29 (6.9)	2 (0.7)
6–11	288	25 (8.3)	30 (10.4)	7 (2.3)
12–23	580	114 (17.3)	39 (6.4)	14 (2.1)
24–35	552	163 (27.5)	19 (4.5)	9 (2.0)
36–47	505	118 (22.9)	8 (1.9)	4 (0.6)
48–59	483	82 (15.3)	8 (1.2)	3 (0.4)
*Gender*				
Male	1407	295 (19.7)	65 (4.4)	21 (1.3)
Female	1313	227 (15.9)	68 (5.1)	18 (1.4)
*Region*				
Western	282	49 (16.6)	11 (3.8)	3 (1.2)
Central	281	63 (22.1)	14 (6.8)	4 (1.1)
Greater Accra	205	20 (9.1)	7 (4.1)	2 (1.0)
Volta	216	41 (19.2)	4 (2.4)	1 (0.5)
Eastern	240	38 (15.3)	9 (3.8)	3 (1.1)
Ashanti	267	38 (14.9)	9 (3.8)	2 (1.5)
Brong Ahafo	316	47 (14.4)	15 (4.2)	2 (0.7)
Northern	430	142 (33.5)	27 (6.5)	12 (2.9)
Upper East	252	35 (15.2)	25 (10.1)	7 (3.2)
Upper West	231	49 (21.9)	12 (4.3)	1 (0.7)

**Table 4:** Correlation between length-for-age *Z*-score (LAZ) and weight-for-length (WLZ) stratified by age group.

	Age (months)						
	0–5	6–11	12–23	24–35	36–47	48–59	0–59
Correlation coefficient (*r*)	−0.332	0.086	0.184	0.101	0.123	−0.032	0.009
Significance (*p* value)	<0.001	0.15	<0.001	0.02	0.006	0.48	0.63
Frequency (*n*)	312	288	580	552	505	483	2720

**Table 5:** Moderation effects of age group on WHZ in predicting HAZ (*N* = 2720).

Model		Unstandardized coefficients		Standardized coefficients	*T*	Sig.	95.0% CI for *β*		Collinearity statistics	
		*B*	Std. error	*β*			Lower bound	Upper bound	Tolerance	VIF
1	(Constant)	−3.10	0.19		−16.054	<0.001	−3.47	−2.72		
	Under five in household	0.16	0.05	0.07	3.001	0.003	0.05	0.26	0.93	1.08
	ANC visits (≥4)	0.03	0.01	0.06	2.44	0.02	0.005	0.05	0.85	1.17
	Sex of child	0.17	0.06	0.07	3.08	0.002	0.06	0.28	1.00	1.00
	Household wealth index (richest)	0.12	0.03	0.13	4.78	<0.001	0.07	0.17	0.64	1.57
	Maternal educational level	0.09	0.04	0.06	2.46	0.01	0.02	0.16	0.67	1.49
	Maternal height (≥160 cm)	0.29	0.03	0.21	9.68	<0.001	0.23	0.35	1.00	1.002

2	(Constant)	−2.82	0.19		−15.21	<0.001	−3.18	−2.46		
	Under five in household	−0.08	0.05	−0.03	−1.53	0.13	−0.18	0.02	0.83	1.20
	ANC visits (≥4)	0.03	0.01	0.06	2.92	0.003	0.01	0.05	0.85	1.17
	Sex of child	0.14	0.05	0.06	2.74	0.006	0.04	0.25	1.00	1.004
	Household wealth index (richest)	0.13	0.02	0.14	5.29	<0.001	0.08	0.17	0.63	1.58
	Maternal educational level	0.05	0.03	0.04	1.39	0.16	−0.02	0.12	0.67	1.50
	Maternal height (≥160 cm)	0.29	0.03	0.21	10.18	<0.001	0.23	0.35	1.00	1.003
	Weight-for-height *Z*-score	0.01	0.03	0.01	0.52	0.60	−0.04	0.06	0.98	1.02
	Age groups of children	−0.42	0.03	−0.30	−13.94	<0.001	−0.48	−0.36	0.88	1.13

3	(Constant)	−2.80	0.18		−15.18	<0.001	−3.16	−2.43		
	Number of children aged under five in household	−0.09	0.05	−0.04	−1.71	0.087	−0.19	0.01	0.83	1.21
	ANC visits (≥4)	0.03	0.01	0.07	2.97	0.003	0.01	0.05	0.85	1.17
	Sex of child	0.14	0.05	0.06	2.71	0.007	0.04	0.24	1.00	1.004
	Household wealth index (richest)	0.13	0.02	0.14	5.42	<0.001	0.08	0.17	0.63	1.58
	Maternal educational level	0.04	0.03	0.03	1.28	0.20	−0.02	0.11	0.67	1.50
	Maternal height (≥160 cm)	0.28	0.03	0.20	10.05	<0.001	0.23	0.34	1.00	1.004
	*Z*-score: weight-for-height *Z*-score	0.09	0.03	0.07	3.05	0.002	0.03	0.15	0.76	1.33
	*Z*-score: age groups of children	−0.43	0.03	−0.31	−14.30	<0.001	−0.49	−0.37	0.88	1.14
	Interaction term	0.15	0.03	0.12	5.39	<0.001	0.10	0.21	0.77	1.31

Dependent variable: height-for-age standard deviation (according to WHO).

**Table 6:** Logistic regressions predicting odds of stunting and wastingamongchildren aged 0–59 months.

Variable	Stunting		Wasting	
	COR (95% CI)	AOR (95% CI)	COR (95% CI)	AOR (95% CI)
*Gender*				
Male	Reference	Not significant in the final model	Reference	Not significant in the final model
Female	0.79 (0.65, 0.96)^*∗*^		1.13 (0.79, 1.60)	
*Birth weight*				
≥2.5 kg	Reference	Reference	Reference	Reference
<2.5 kg	2.51 (1.75, 3.62)^*∗∗∗*^	2.68 (1.67, 4.31)^*∗∗∗*^	2.17 (1.20, 3.92)^*∗*^	2.00 (1.10, 3.64)^*∗*^
*Frequency of ANC attendance*				Not significant in the final model
≥4	Reference	Reference	Reference	
0–3	2.75 (1.29, 2.37)^*∗∗∗*^	2.84 (1.63, 4.95)^*∗∗∗*^	1.46 (0.89, 2.38)	
*Maternal education*		Not significant in the final model		Not significant in the final model
Higher	Reference		Reference	
No education	6.38 (2.56, 15.87)^*∗∗∗*^		3.00 (0.72, 12.46)	
Primary	4.60 (1.83, 11.58)^*∗∗*^		2.15 (0.50, 9.24)	
Secondary	2.82 (1.13, 7.06)^*∗*^		2.11 (0.50, 8.81)	
*Household wealth index*				
Richest		Reference		Reference
Poorest	4.04 (2.62, 6.25)^*∗∗∗*^	2.36 (1.29, 4.30)^*∗∗*^	2.25 (1.17, 4.33)^*∗*^	2.33 (1.12, 4.87)^*∗*^
Poorer	3.82 (2.43, 5.9)^*∗∗∗*^	1.78 (0.94, 3.38)	1.34 (0.65, 2.77)	1.67 (0.72, 3.86)
Middle	2.488 (1.56, 3.98)^*∗∗∗*^	1.61 (0.86, 3.03)	0.76 (0.34, 1.73)	0.68 (0.26, 1.76)
Richer	2.085 (1.27, 3.42)^*∗∗*^	1.79 (0.95, 3.37)	1.62 (0.77, 3.41)	1.40 (0.61, 3.21)
*Child is wasted?*				
No	Reference	Reference	Reference	Reference
Yes	1.81 (1.23, 2.66)^*∗*^	2.00 (1.04, 3.88)^*∗*^	Not applicable	Not applicable
*Parity*				Not significant in the final model
1-2	0.56 (0.44, 0.71)^*∗∗∗*^	0.47 (0.31, 0.74)^*∗∗*^	0.91 (0.59, 1.40)	
3-4	0.74 (0.59, 0.93)^*∗*^	0.67 (0.43, 1.74)	1.09 (0.71, 1.68)	
>4	Reference			
*Maternal height (cm)*				Not significant in the final model
At least 160	Reference	Reference		
<145	5.66 (2.54, 12.61)^*∗∗∗*^	4.88 (1.15, 20.65)^*∗*^	1.64 (0.38, 7.12)	
145–149	3.75 (2.56, 5.49)^*∗∗∗*^	3.18 (1.54, 6.56)^*∗∗*^	1.02 (0.46, 2.27)	
150–154	2.02 (1.54, 2.64)^*∗∗∗*^	1.81 (1.10, 2.95)^*∗*^	0.75 (0.43, 1.30)	
155–159	1.50 (1.19, 1.89)^*∗∗*^	1.70 (1.14, 2.54)^*∗∗*^	1.02 (0.69, 1.51)	
*Age of child (months)*				
0–5		Reference		Reference
6–11	1.39 (0.75, 2.56)	1.84 (0.72, 4.73)	1.14 (0.66, 1.94)	0.89 (0.44, 1.80)
12–23	3.57 (2.17, 5.87)^*∗∗∗*^	4.58 (2.0, 10.50)^*∗∗∗*^	0.70 (0.43, 1.16)	0.70 (0.37, 1.33)
24–35	6.12 (3.75, 9.97)^*∗∗∗*^	9.76 (4.29, 22.22)^*∗∗∗*^	0.35 (0.19, 0.63)^*∗∗*^	0.35 (0.17, 0.75)^*∗∗*^
36–47	4.45 (2.71, 7.32)^*∗∗∗*^	4.92 (2.01, 12.06)^*∗∗∗*^	0.16 (0.07, 0.35)^*∗∗∗*^	0.07 (0.02, 0.31)^*∗∗∗*^
48–59	2.99 (1.79, 4.98)^*∗∗∗*^	3.25 (2.17, 5.87)^*∗*^	0.16 (0.07, 0.36)^*∗∗∗*^	0.10 (0.02, 0.42)^*∗∗*^
*Type of place of residence*		Not significant in the final model		
Urban			Reference	Reference
Rural	1.74 (1.41, 2.13)^*∗∗∗*^		1.43 (0.98, 2.07)	1.62 (1.01, 2.58)^*∗*^
*Toilet facility*		Not significant in the final model		Not significant in the final model
No facility	Reference		Reference	
Flush	0.41 (0.29, 0.60)^*∗∗∗*^		0.75 (0.42, 1.33)	
Pit latrine	0.70 (0.57, 0.86)^*∗∗*^		0.65 (0.45, 0.95)^*∗*^	
*Under 5 in household*		Not significant in the final model		Not significant in the final model
0-1	Reference		Reference	
2-3	1.27 (1.04, 1.55)^*∗*^		1.10 (0.7, 1.59)	
≥4	2.55 (1.65, 3.94)^*∗∗∗*^		1.76 (0.81, 3.81)	

^*∗*^Significant at *p* < 0.05; ^*∗∗*^significant at *p* < 0.01; and ^*∗∗∗*^significant at *p* < 0.001.

AOR (95% CI): adjusted odds ratio at 95% confidence level.

COR (95% CI): crude/unadjusted odds ratio at 95% confidence level.

## References

[1] Black R. E., Allen L. H., Bhutta Z. A. (2008). Maternal and child undernutrition: global and regional exposures and health consequences. *The Lancet*.

[2] WHO (2013). *Long-Term Effects of Breastfeeding: A Systematic Review*.

[3] Shrimpton R., Victora C. G., de Onis M., Lima R. C., Blössner M., Clugston G. (2001). Worldwide timing of growth faltering: implications for nutritional interventions. *Pediatrics*.

[4] WHO (2013). *Essential Nutrition Actions: Improving Maternal, Newborn, Infant and Young Child Health and Nutrition*.

[5] Sundaram M. E., Labrique A. B., Mehra S. (2013). Early neonatal feeding is common and associated with subsequent breastfeeding behavior in rural Bangladesh. *Journal of Nutrition*.

[6] WHO (2012). Newborn reducing mortality. *Fact Sheet*.

[7] Save The Children

[8] WHO (2010). *Nutrition Landscape Information System (NLIS) Country Profile Indicators: Interpretation Guide*.

[9] Saaka M., Iddrisu M. (2014). Patterns and determinants of essential newborn care practices in rural areas of northern Ghana. *International Journal of Population Research*.

[10] Mamabolo R. L., Alberts M., Steyn N. P., Delemarre-Van De Waal H. A., Levitt N. S. (2005). Prevalence and determinants of stunting and overweight in 3-year-old black South African children residing in the Central Region of Limpopo Province, South Africa. *Public Health Nutrition*.

[11] Legesse M., Demena M., Mesfin F., Haile D. (2014). Prelacteal feeding practices and associated factors among mothers of children aged less than 24 months in Raya Kobo district, North Eastern Ethiopia: a cross-sectional study. *International Breastfeeding Journal*.

[12] UNICEF (2012). *Levels & Trends in Child Mortality Report*.

[13] de Onis M., Blössner M., Borghi E. (2010). Global prevalence and trends of overweight and obesity among preschool children. *The American Journal of Clinical Nutrition*.

[14] Ghana Statistical Service (GSS) (2015). *Ghana Demographic and Health Survey 2014*.

[15] WHO (2006). *Who Child Growth Standards: Length/Height-for-Age, Weight-for-Age, Weight-for-Length, Weight-for-Height and Body Mass Index-for-Age: Methods and Development*.

[16] Rustein S. O., Johnson K. (2004). *The DHS Wealth Index*.

[17] Nandy S., Irving M., Gordon D., Subramanian S. V., Smith G. D. (2005). Poverty, child undernutrition and morbidity: new evidence from India. *Bulletin of the World Health Organization*.

[18] McDonald C. M., Olofin I., Flaxman S. (2013). The effect of multiple anthropometric deficits on child mortality: meta-analysis of individual data in 10 prospective studies from developing countries. *American Journal of Clinical Nutrition*.

[19] Khara T., Dolan C. (2014). *Technical Briefing Paper: The Relationship Between Wasting and Stunting: Policy, Programming and Research Implications*.

[20] Richard S. A., Black R. E., Gilman R. H. (2012). Wasting is associated with stunting in early childhood. *Journal of Nutrition*.

[21] Martorell R., Blaxter K., Waterlow J. C. (1985). Child growth retardation: a discussion of its causes and its relationship to health. *Nutritional Adaptation in Man*.

[22] Victora C. G. (1992). The association between wasting and stunting: an international perspective. *Journal of Nutrition*.

[23] Mattar C. N., Chong Y.-S., Chan Y.-S. (2007). Simple antenatal preparation to improve breastfeeding practice: a randomized controlled trial. *Obstetrics and Gynecology*.

[24] Frongillo E. A., de Onis M., Hanson K. M. (1997). Socioeconomic and demographic factors are associated with worldwide patterns of stunting and wasting of children. *Journal of Nutrition*.

[25] Costello A. M. (1989). Growth velocity and stunting in rural Nepal. *Archives of Disease in Childhood*.

[26] Maleta K., Virtanen S. M., Espo M., Kulmala T., Ashorn P. (2003). Seasonality of growth and the relationship between weight and height gain in children under three years of age in rural Malawi. *Acta Paediatrica*.

[27] Nabarro D., Howard P., Cassels C., Pant M., Wijga A., Padfield N., Waterlow J. (1988). The importance of infections and environmental factors as possible determinants of growth retardation in children. *Linear Growth Retardation in Developing Countries*.

[28] Brown K. H., Black R. E., Becker S. (1982). Seasonal changes in nutritional status and the prevalence of malnutrition in a longitudinal study of young children in rural Bangladesh. *The American Journal of Clinical Nutrition*.

[29] Dewey K. G., Hawck M. G., Brown K. H., Lartey A., Cohen R. J., Peerson J. M. (2005). Infant weight-for-length is positively associated with subsequent linear growth across four different populations. *Maternal and Child Nutrition*.

[30] Walker S. P., Golden M. H. N. (1988). Growth in length of children recovering from severe malnutrition. *European Journal of Clinical Nutrition*.

[31] Doherty C. P., Sarkar M. A. K., Shakur M. S., Ling S. C., Elton R. A., Cutting W. A. (2001). Linear and knemometric growth in the early phase of rehabilitation from severe malnutrition. *British Journal of Nutrition*.

[32] Karsenty G. (2006). Convergence between bone and energy homeostases: leptin regulation of bone mass. *Cell Metabolism*.

[33] Gat-Yablonski G., Ben-Ari T., Shtaif B. (2004). Leptin reverses the inhibitory effect of caloric restriction on longitudinal growth. *Endocrinology*.

[34] Popkin B. M., Richards M. K., Montiero C. A. (1996). Stunting is associated with overweight in children of four nations that are undergoing the nutrition transition. *Journal of Nutrition*.

[35] Golden M. H. (2009). Proposed Recommended Nutrient densities for moderately malnourished children. *Food and Nutrition Bulletin*.

[36] Briend A., Khara T., Dolan C. (2015). Wasting and stunting—similarities and differences: policy and programmatic implications. *Food and Nutrition Bulletin*.

[37] Martorell R., Young M. F. (2012). Patterns of stunting and wasting: potential explanatory factors. *Advances in Nutrition*.

[38] Rahman M. M. (2015). Is Unwanted birth associated with child malnutrition in Bangladesh?. *International Perspectives on Sexual and Reproductive Health*.

[39] Doak C., Adair L., Bentley M., Fengying Z., Popkin B. (2002). The underweight/overweight household: an exploration of household sociodemographic and dietary factors in China. *Public Health Nutrition*.

[40] Hong R., Banta J. E., Betancourt J. A. (2006). Relationship between household wealth inequality and chronic childhood under-nutrition in Bangladesh. *International Journal for Equity in Health*.

[41] Petrou S., Kupek E. (2010). Poverty and childhood undernutrition in developing countries: a multi-national cohort study. *Social Science and Medicine*.

[42] Neupane S., Nwaru B. I. (2014). Impact of prenatal care utilization on infant care practices in Nepal: a national representative cross-sectional survey. *European Journal of Pediatrics*.

[43] Swigonski N. L., Skinner C. S., Wolinsky F. D. (1995). Prenatal health behaviors as predictors of breast-feeding, injury, and vaccination. *Archives of Pediatrics and Adolescent Medicine*.

[44] Medhin G., Hanlon C., Dewey M. (2010). Prevalence and predictors of undernutrition among infants aged six and twelve months in Butajira, Ethiopia: The P-MaMiE Birth Cohort. *BMC Public Health*.

[45] Christian P., Lee S. E., Donahue Angel M. (2013). Risk of childhood undernutrition related to small-for-gestational age and preterm birth in low- and middle-income countries. *International Journal of Epidemiology*.

[46] Maleta K., Virtanen S. M., Espo M., Kulmala T., Ashorn P. (2003). Childhood malnutrition and its predictors in rural Malawi. *Paediatric and Perinatal Epidemiology*.

[47] Khara T. N., Mates E. (2013). Maternal nutrition in emergencies: summary of the state of play and key gaps.

[48] Fernandez I. D., Himes J. H., de Onis M. (2002). Prevalence of nutritional wasting in populations: building explanatory models using secondary data. *Bulletin of the World Health Organization*.

[49] Mehnaz S., Khan Z., Khalique N., Amir A. (2010). Place of delivery vis-à-vis exclusive breastfeeding and morbidities in young children. *Indian Journal of Preventive & Social Medicine*.

[50] Lassi Z. S., Majeed A., Rashid S., Yakoob M. Y., Bhutta Z. A. (2013). The interconnections between maternal and newborn health-evidence and implications for policy. *Journal of Maternal-Fetal and Neonatal Medicine*.

[51] Pongou R., Ezzati M., Salomon J. A. (2006). Household and community socioeconomic and environmental determinants of child nutritional status in Cameroon. *BMC Public Health*.

[52] Özaltin E., Hill K., Subramanian S. V. (2010). Association of maternal stature with offspring mortality, underweight, and stunting in low- to middle-income countries. *The Journal of the American Medical Association*.

[53] Birmeta K., Dibaba Y., Woldeyohannes D. (2013). Determinants of maternal health care utilization in Holeta town, central Ethiopia. *BMC Health Services Research*.

[54] Keller W., Waterlow J. C. (1988). The epidemiology of stunting. *Linear Growth Retardation in Developing Countries*.

[55] Huttly S. R. A., Victora C. G., Barros F. C., Teixeira A. M. B., Vaughan J. P. (1991). The timing of nutritional status determination: implications for interventions and growth monitoring. *European Journal of Clinical Nutrition*.

